# Simple cryopreservation protocol for *Luffa* pollen: enhancing breeding efficiency

**DOI:** 10.3389/fpls.2023.1268726

**Published:** 2023-10-27

**Authors:** Arvind Nagar, Ravi Gowthami, Amish Kumar Sureja, Anilabha Das Munshi, Manjusha Verma, Awani Kumar Singh, Niharika Mallick, Jogendra Singh, Subhash Chander, Muthusamy Shankar, Pooja Pathania, Subramani Rajkumar

**Affiliations:** ^1^ Krishi Vigyan Kendra (KVK), Jhalawar, Agricultural University, Kota, India; ^2^ Division of Germplasm Conservation, ICAR-National Bureau of Plant Genetic Resources, New Delhi, India; ^3^ Division of Vegetable Science, ICAR-Indian Agricultural Research Institute, New Delhi, India; ^4^ Division of Genomic Resources, ICAR-National Bureau of Plant Genetic Resources, New Delhi, India; ^5^ CPCT, ICAR-Indian Agricultural Research Institute, New Delhi, India; ^6^ Division of Genetics, ICAR-Indian Agricultural Research Institute, New Delhi, India; ^7^ Division of Plant Genetic Resources, The Graduate School, Indian Agricultural Research Institute, Indian Council of Agricultural Research (ICAR), New Delhi, India

**Keywords:** cryopreservation, incubation temperature, long-term storage, *Luffa*, pollen, storage temperature, ultra-low freezing

## Abstract

This study aimed to develop a long-term pollen storage protocol for *Luffa* species (*L. acutangula*, *L. cylindrica*, *L. echinata*, and *L. graveolens*) and assess its potential for crop improvement. The optimal medium for *in vitro* pollen germination varied by species, with Brewbaker and Kwack (BK) medium with 10% sucrose suitable for *L. acutangula, L. cylindrica*, and *L. echinata*, and BK medium with 3% sucrose ideal for *L. graveolens*. Overestimation in staining tests compared to *in vitro* pollen germination was observed. The best results for cryopreservation were achieved with desiccation periods of 20, 30, and 40 min, maintaining moisture content between 14.04% and 18.55%. Pollen viability was negatively correlated with storage temperature (25, 4, and −20°C) and duration. Cryopreserved pollen at −196°C exhibited the highest viability over a prolonged period (2 months) and was comparable to fresh pollen in terms of germination, ovule fertilization, and fruit and seed set. This study presents a simple and reproducible pollen cryopreservation protocol applicable across *Luffa* species, facilitating long-term storage and its use in crop improvement efforts.

## Introduction

The genus *Luffa* encompasses nine species, namely, seven wild species [*L. tuberosa* Roxb., *L. graveolens* Roxb. (var. *longistyla*), *L. astorii* Svans, *L. echinata* Roxb., *L. quinquefida* (Hook and Arn), *L. saccata*, and *L. umbellata* Roem] and two cultivated species (*L. acutangula* and *L. cylindrica*) within the Cucurbitaceae family (Dhillon et al., 2017). *L*. *acutangula* and *L*. *cylindrica* are annual vines bearing hermaphrodite, staminate, or pistillate flowers (Kumari et al., 2019). Ridge gourd, a member of the *Luffa* genus, exhibits diverse sex forms, including androecious, andromonoecious, gynoecious, gynomonoecious, monoecious, and hermaphrodite (Choudhary et al., 2011). *Luffa* species are highly valued for their nutritional content (carotenoids, amino acids, proteins, lipids, alkaloids, flavonoids, etc.) and have a significant presence in the Asian vegetable market, where they are consumed in boiled and fried forms (Badgujar and Patil, 2008; Partap et al., 2012).

Interspecific hybridization in *Luffa* holds the potential for transferring novel genes related to biotic and abiotic stress resistance. However, this process comes with its challenges, including (a) asynchronization due to differences in sex forms among species, and (b) temporal variations in flowering. In this study, both monoecious and dioecious species were utilized. *L. acutangula*, *L. cylindrica*, and *L. graveolens* are monoecious vines, bearing separate male and female flowers on the same plants (Jamwal and Sharma, 2017), while *L. echinata* is dioecious and bears male and female flowers on the different plants. The time gap between the first staminate flower’s appearance at the lower node and the first female flower on the middle node varies among species (13–15 days in *L. cylindrica*, 16–17 days in *L. acutangula*, and 12–15 days in *L. graveolens*). Additionally, there are temporal differences in flower opening, such as evening anthesis in *L. acutangula* compared to early morning anthesis in *L. cylindrica*, *L. echinata*, and *L. graveolens*. Recent advancements have utilized male sterility (cytoplasmic genetic male sterility-CGMS) effectively in *Luffa* hybrid development (Sharma et al., 2019; Karmakar et al., 2022). However, maintaining the “B” line (in CGMS) separately is labor- and space-intensive and often faces asynchronization with the female line. To address these challenges, it is essential to ensure a consistent supply of pollen throughout hybridization activities to facilitate a sufficient number of pollinations and better exploit the diversity present in this crop.

Pollen storage plays a crucial role in overcoming these challenges. Pollen storage methods range from room temperature storage (25°C) to low-temperature storage in household refrigerators (4°C), freezers (−20°C), and deep freezers (−80°C), with options for storing fresh, desiccated, vacuum- or freeze-dried pollen. However, pollen can only be stored for short- to medium-term periods (hours to days to weeks to months) at these temperatures (Fayos et al., 2022; Wu et al., 2022; Shankar et al., 2023). Cryopreservation is the storage of pollen at ultra-low temperature in liquid nitrogen (LN) either in the liquid phase (−196°C) or in the vapor phase (−150 to −180°C) retaining its original viability and offers long-term storage (Rajasekharan and Rohini, 2023). Thus, in the present study, four species of *Luffa* genus, namely, *L. acutangula*, *L. cylindrica*, *L. echinata*, and *L. graveolens*, were utilized for pollen cryopreservation studies with the objectives of (a) standardizing the optimum *in vitro* pollen germination medium; (b) identifying the reliable pollen viability assessment method; (c) studying the effect of different storage temperatures and duration on pollen viability; (d) identifying the optimum moisture content, desiccation period, and thawing temperature for cryopreservation; and (e) standardizing the species-wide pollen cryopreservation protocol.

## Materials and methods

### Plant material

Four species of the *Luffa* genus, namely, *L. acutangula* (Pusa Nutan, Swarna Uphar), *L. cylindrica* (Pusa Supriya, Pusa Chikni), *L. echinata* (IC618051), and *L. graveolens* (PKS-4, PKS-20), were utilized for pollen cryopreservation studies. Seeds of wild *Luffa* species (*L. echinata* and *L. graveolens*) were obtained from the ICAR-National Bureau of Plant Genetic Resources in 2016. Wild species and cultivars of cultivated species have been maintained through self-pollination at the Division of Vegetable Science, Indian Agricultural Research Institute (IARI), New Delhi. The seeds were stored in desiccators containing silica gel at room temperature until use. The plants were grown in an insect-proof nethouse at the Centre for Protected Cultivation Technology, IARI, New Delhi, India (28°37′35"N, 77°09′35"E, altitude 250 m above sea level). The soil in the polyhouse was sandy loam to loamy with a pH of 7.5. Fifteen plants per genotype were planted in mid-February 2023 and were maintained following the standard package of practices for *Luffa* crop cultivation to ensure healthy growth.

### Anthesis, anther dehiscence, and pollen collection

Anthesis and anther dehiscence were recorded by tagging 10 flowers in each replication, with each replication consisting of five plants. For pollen collection, freshly opened or nearly opened flowers were collected from the polyhouse during peak flowering period in May 2023 between 5:00 and 6:00 PM in *L. acuntagula* and between 8:00 and 9:00 AM in *L. cylindrica*, *L. echinata*, and *L. graveolens* due to the nocturnal and diurnal nature of flowering. These flowers were placed in properly labeled butter paper bags. In laboratory, petals were carefully separated, and the dehisced anthers were tapped to release pollen into a clean glass Petri plate lined with butter paper. Debris was removed, and collected pollens were pooled for each genotype, and subjected to viability assessment, and storage.

### Pollen viability tests

Pollen viability was evaluated in *Luffa* species using staining methods, specifically potassium iodide (2%) and acetocarmine (2%). In addition, *in vitro* germination was also employed. Each experiment was organized in a Completely Randomized Design (CRD) with three replications for each species. In each replication, 10 microscopic field views were observed, with each replication containing a minimum of 500 to 600 pollen grains.

#### *In vitro* germination test

In the *in vitro* germination tests, Brewbaker and Kwack (BK) medium was used. The BK medium is composed of boric acid (100 mg/L), calcium nitrate (300 mg/L), magnesium sulfate (200 mg/L), and potassium nitrate (100 mg/L) (Sigma^®^) (Brewbaker and Kwack, 1963), and pH was adjusted to 5.84. In this experiment, (a) the effect of BK medium (Brewbaker and Kwack medium) with different sucrose (Himedia^®^) concentrations (0%, 5%, 10%, 15%, 20%, 25%, and 30%) and (b) the effect of incubation temperatures (10, 15, 20, and 30°C) on the *in vitro* germination of fresh pollen were assessed across different *Luffa* species. Freshly harvested pollens were applied to a glass slide (Blue star^®^, 75 mm long x 25 mm wide, 1.35 mm thickness) containing a 2.0-μl drop of BK medium with different concentrations of sucrose and covered with a cover slip. The slides were then placed in a glass Petri plate (15 cm diameter; Borosil^®^) containing moist filter paper to maintain 70%–80% humidity at different incubation temperatures for 2 h. Germination percentages were calculated by dividing the number of germinated pollens by the total number of pollens in each field view and under a compound microscope (Carl Zeiss AX10 Microscope; 10×). Pollen grains were considered germinated and viable if the length of the tube exceeded the diameter of the grain.

#### Potassium iodide test (KI) and acetocarmine (AC) test

Pollen viability was also assessed using a potassium iodide (KI) (2%) and acetocarmine (AC) (2%) solution. A drop (2 μl) of solution was placed on a micro slide, and pollen grains were evenly spread in the solution using a needle. Slides were then covered and incubated for 15 min at room temperature (25 ± 2° C) for KI and 30 min for AC staining. Subsequently, stained and unstained pollen grains were counted under a compound microscope (Carl Zeiss AX10 Microscope; 10×). Black-stained (KI) and bright red-stained (AC) pollen grains were considered viable, while unstained pollen grains were categorized as non-viable (Shivanna and Tandon, 2014).

### Determination of moisture content, desiccation period, and thawing temperature

The experiments were laid out in a CRD with three replications for each species, and in each replication, 10 microscopic field views were taken. Every replication had a minimum of 500–600 pollen grains.

#### Fresh pollen moisture content

Fresh pollen moisture content (MC) was assessed in all seven genotypes of four *Luffa* species. To determine moisture content, fresh pollen grains were collected from each species in an aluminum cup. The pollen grain was oven dried at 105°C for 2 h (Ren et al., 2019) and the dry weight of pollen grains was recorded. The moisture content was calculated using the formula MC = [(FW − DW)/(DW − TW)], where MC is pollen moisture content, FW is fresh weight, DW is dry weight, and TW is the tare weight, the empty weight of the aluminum cup.

#### Desiccation period

Fresh pollen was placed in aluminum foil cups and incubated in a laminar air flow cabinet for air drying at intervals of 0, 10, 20, 30, 40, 50, 60, and 70 min. The moisture content at each desiccation period was calculated using the equation MC = [(FW − DeW)/(DeW − TW)], where DeW is the weight of the pollen grains after desiccation. To establish the optimum moisture content and desiccation for cryopreservation, both fresh and desiccated pollen (desiccated for various time intervals, including 0, 10, 20, 30, 40, 50, 60, and 70 min) were packed in aluminum pouches. These pouches were then placed inside cryovials (1.8 mL). Subsequently, the cryovials were arranged in polycarbonate freezer boxes (Thermo Fisher Scientific^®^, Roskilde, Denmark) and immediately immersed in LN cryotanks (Air Liquide, France). Pollen viability was assessed for both fresh and desiccated pollen after 12 h of cryopreservation, followed by thawing at room temperature for 20 min.

#### Thawing temperature

After 12 h of cryostorage, cryovials were removed from the cryotanks and thawed at different temperatures; (a) thawing at a room temperature of 25°C ( ± 2°C) for 20 min, (b) thawing under running water at 20°C ( ± 5°C) for 10 min, (c) thawing in water held at 35°C for 5 min with constant slow stirring, (d) thawing under 10°C for 5 min, and (e) thawing under 10°C for 2 min. followed by thawing at 35°C for 3 min.

### Pollen storage regimes

Fresh and desiccated pollen from four *Luffa* species, namely, *L. acutangula* (Pusa Nutan), *L. cylindrica* (Pusa Chikni), *L. echinata* (IC618051), and *L. graveolens* (PKS-4), were collected and stored under four temperature regimes; 25, 4, −20, and −196°C, under dark conditions. For storage, pollen grains were packed in aluminum pouches and placed inside cryovials (1.8 mL), which were then organized within a cryobox. The cryobox was kept in the dark and covered with a sheet of aluminum foil to maintain light exclusion. The various storage temperatures were achieved by placing the cryobox in the laboratory at 25°C, in a refrigerator at 4°C, and in a deep freezer at −20°C. Cryopreservation was achieved by immersing the cryovials containing pollen in LN cryotanks (Air Liquide, France). The viability of stored pollen grains was assessed using an optimized medium through the *in vitro* pollen germination method. The viability of the stored pollen grains was monitored at different time intervals, including 0 h, 1 h, 3 h, 5 h, 9 h, 12 h, 24 h, 1 week, 2 weeks, and 2 months.

### Fertilizing ability of cryopreserved pollen

#### Fluorescent microscopy

Cryopreserved pollen was delicately applied to the stigmas of bagged flowers of *L. acutangula* (Pusa Nutan) using a soft camel brush and then bagged. Fresh pollen of the same genotype was used as a control. Pollinated pistils were fixed in FAA/Carnoy’s solution (a solution containing 5 mL of formalin, 5 mL of acetic acid, and 90 mL of 70% ethanol) (Sun et al., 2010) at intervals of 12, 24, and 48 h after pollination (HAP) with fresh and cryopreserved pollen. The fixed pistils were stored at 4°C until assessment. The germination of pollen grains on stigmas was examined using five buds each for fresh and cryopreserved pollen, employing fluorescence microscopy (Leica DM6 B, Germany). Fixed and stored pistils were rinsed twice in distilled water, and buds were softened by soaking in 6N NaOH solution overnight. The pistils were subsequently rinsed in 50 mM potassium phosphate buffer (pH 7.5) and then stained for 12 h in the dark with 0.05% aniline blue (prepared in 50 mM potassium phosphate buffer) (Schiøtt et al., 2004). After sufficient staining, the dissected pistils were mounted on glass slides in the same staining solution and covered with a cover slip. Pollen germination was visualized on the stigmatic surface under UV light (390–420 nm) using a fluorescence microscope (Leica DM6 B, Germany), and images were captured. The pistils were observed for pollen germination on the stigma and pollen tube entry to the embryo sac through the micropyle and ovule.

#### Field pollinations

**Figure 1:**
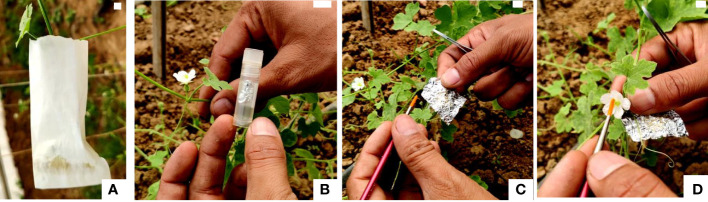
Fertilizing ability of cryopreserved pollen using field pollinations. **(A)** Bagging of female flower bud before a day of anthesis with butter paper cover. **(B)** Cryovials with cryopreserved pollen. **(C)** Removal of aluminum envelope with pollen from the cryovial, gentle unfolding of aluminum envelope, and capturing of pollen with soft camel brush. **(D)** Dusting of cryopreserved pollen on receptive stigma using soft camel brush (Scale bar = 1 cm).

For field pollinations, flower buds about to open in the evening in nocturnal species and in the morning in diurnal species were covered with butter paper bags (**Figure 1A**). The following day, these buds were pollinated with fresh and cryopreserved pollen (**Figures 1B–D**). All pollinated flowers were covered with small butter paper covers and labeled with tags tied to the pedicel of crossed flowers. Data were recorded on various parameters including fruit set (%), fruit length (cm), fruit width (cm), average fruit weight (g), number of seeds per fruit, seed length (mm), and seed width (mm). These experiments were conducted during the first and second weeks of May.

### Statistical analysis

All the experiments were conducted in CRD with three replications. Data were presented as the mean with standard error. For normality, the data underwent an arcsine transformation. One-way ANOVA was performed, followed by Duncan’s Multiple Range Test (DMRT) for comparison between treatments (*p ≤* 0.05). Data analysis was carried out using the SPSS version 22.0 statistical software, and graphs were prepared using Microsoft Excel 2010.

## Results

### Anthesis and anther dehiscence

#### Luffa acutangula

*L. acutangula* is a monoecious plant, with male flowers differentiating and opening first, primarily on lower nodes. Subsequently, female flowers were open amid a mixture of staminate and pistillate flowers that develops simultaneously on the central nodes of the vines as they reach a height of 2 m. Male flowers are borne on pedunculate racemes, whiles female flowers appear solitarily in the axils of leaves. Anthesis of female flower at upper pistillate nodes overlaps with the anthesis of male flower at lower and middle nodes, whereas the anthesis of female flower at middle nodes synchronizes with the anthesis of male flower at lower staminate nodes. *L. acutangula* blooms in the evening, initiating between 5:30 and 6:00 PM and closing by the following day at 5:00 AM, with complete closure occurring between 6:00 and 7:00 AM. Both male and female flowers remain open throughout the night, with the female flowers closing half an hour later than the male flowers. Anther dehiscence begins approximately half an hour before anthesis, between 5:00 and 6:00 PM and is completed by 10:30 to 12:00 PM.

#### Luffa cylindrica

*L. cylindrica* is also monoecious, with male flowers differentiating and opening first, mainly on lower nodes. As the vine grows to a height of 3 m, the opening of male flowers at lower staminate nodes coincides with the opening of female flowers at middle nodes. The anthesis of male flowers at the lower and intermediate nodes overlaps with the anthesis of female flowers at the top nodes in the later stages of vine developmental*. L. cylindrica* is a diurnal plant, with flower opening commencing between 05:00 and 06:00 AM and ending in the evening between 05:00 and 06:00 PM. Female flowers close approximately 1 h later than male flowers. Anther dehiscence begins about an hour before flower opening, between 4:00 and 6:00 AM and is fully complete by 8:00 to 10:00 AM.

#### Luffa graveolens

*L. graveolens* produces separate male and female flowers on the same plant, making it monoecious. The flowers are typically formed in the leaf axils or at the ends of the vine branches. Occasionally, hermaphrodite buds were observed at a later stage of plant growth. *L. graveolens* tends to begin flowering after the 12th to 15th node. Male flowers are borne in clusters with long peduncles and appear first on the lower nodes. Each male flower typically consists of a yellow corolla with five petals surrounding a central column of stamens that produces pollen. Female flowers are solitary and have shorter peduncles, featuring a small ovary at the base covered with small spines. The opening of male flowers at the lower and intermediate nodes synchronizes with the opening of female flowers at the top nodes during later vine development*. L. graveolens* is a diurnal plant, with flower opening initiated between 05:00 and 06:00 AM and flowering ceasing in the evening between 07:00 and 09:00 PM. Anther dehiscence begins an hour before flower opening, between 4:00 and 5:00 AM and is fully complete by 8:00 to 9:00 AM.

#### Luffa echinata

*L. echinata* produces separate male and female flowers on different plants, making it dioecious. The flowers are typically formed in the leaf axils or at the ends of the vine branches. Male flowers typically appear in clusters with long peduncles, featuring a white corolla with five petals and a central column of stamens producing pollen. Female flowers are solitary and have small ovaries at the base, covered with small soft bristles, and they are borne on short peduncles. *L. echinata* usually begins flowering after the 6 to 9th node. *L. echinata* is diurnal, with flower opening initiated between 05:00 and 06:00 AM and flower dropping by the evening, between 07:00 and 08:00 P.M. Anther dehiscence begins about an hour before flower opening between 4:00 and 5:00 AM and is fully complete by 9:00 to 10:00 AM.

### Pollen viability

**Figure 2:**
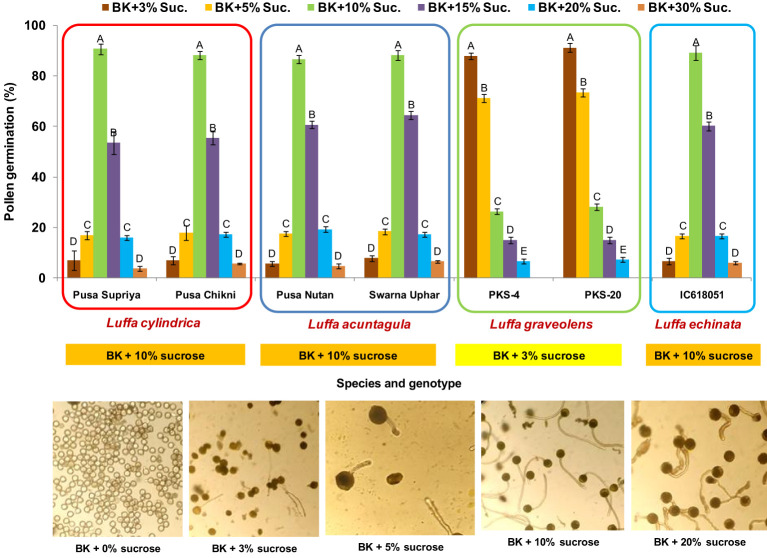
Standardization of *in vitro* pollen germination medium for *Luffa* species. Data presented in vertical bars represent mean ± SE. Significant differences (*P* ≤ 0.05) in pollen germination are presented by different alphabets analyzed by Duncan’s Multiple Range Test. The optimum *in vitro* pollen germination medium is provided in the orange and yellow boxes below each species. Representative images for pollen germination at different sucrose concentrations are provided for *L. cylindrica*.

**Figure 3:**
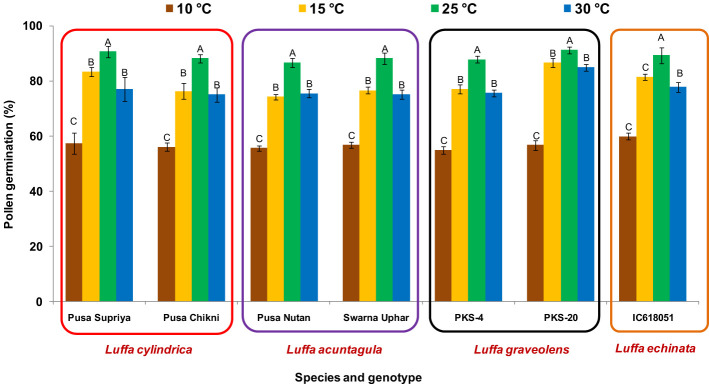
Effect on incubation temperature on pollen viability in *Luffa* species. Data presented in vertical bars represent mean ± SE. Significant differences (*P* ≤ 0.05) in pollen germination are presented by different alphabets analyzed by Duncan’s Multiple Range Test.

**Figure 4:**
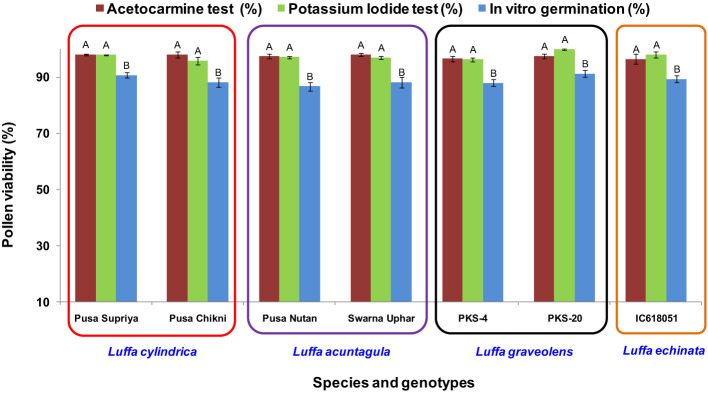
Pollen viability assessment using staining (acetocarmine and potassium iodide) and *in vitro* pollen germination in *Luffa* species. Data presented in vertical bars represent mean ± SE. Significant differences (*P* ≤ 0.05) in pollen viability are presented by different alphabets analyzed by Duncan’s Multiple Range Test.

**Figure 5:**
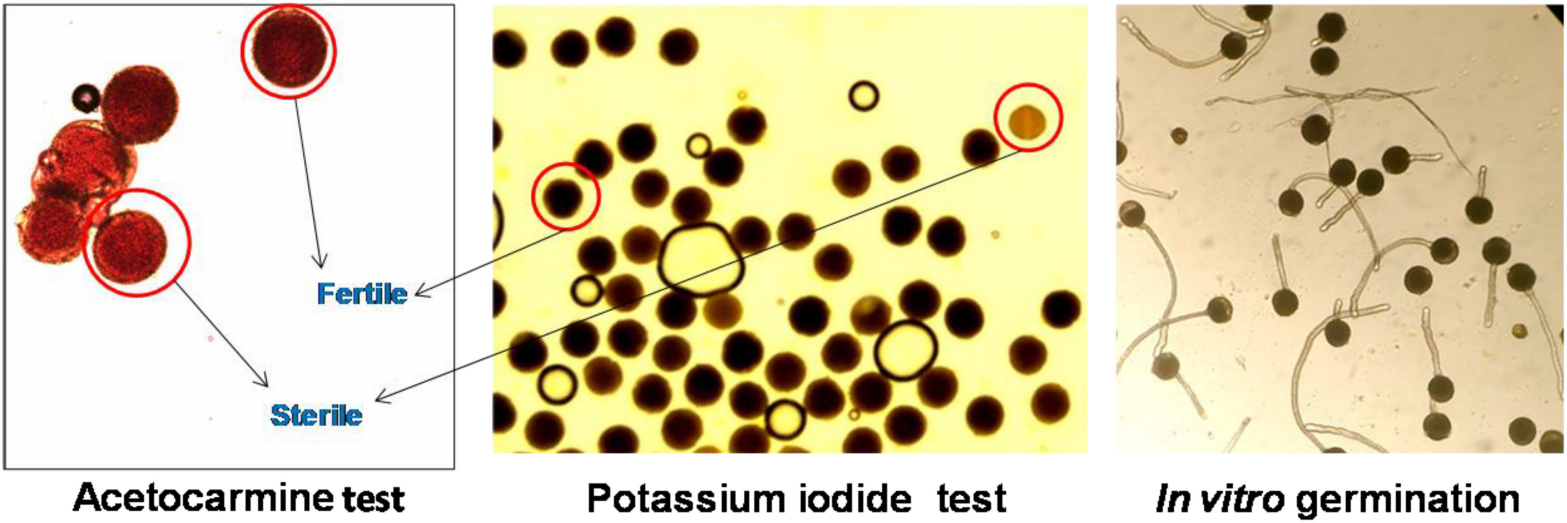
Representative images of pollen viability assessment using different methods in *Luffa echinata*.

In all four species tested, no pollen germination was observed on the control medium (only water) and BK medium without sucrose. Among the tested sucrose concentrations, BK medium with 10% sucrose consistently reported significantly (*p ≤* 0.05) higher fresh pollen germination in *L. cylindrica* (88.15% to 90.61%), *L. acutangula* (86.66% to 88.17%), and *L. echinata* (89.29%). The pollen germination percentage was steadily increased up to 10% sucrose but declined at higher concentrations of sucrose (15%, 20%, and 30%). In contrast, *L. graveolens* showed contradictory results, with the highest pollen germination percentage (87.84% to 91.19%) observed on BK medium with 3% sucrose, followed by 5% sucrose (71.14% to 73.49%) (**Figure 2**). For all tested species, the optimum incubation temperature for pollen germination was 25°C with the highest pollen germination (average of all species: 88.85%) (**Figure 3**). Pollen germination was significantly lower at 10°C of incubation (average of all species: 56.75%), while no significant difference was found at 15°C and 30°C incubation temperatures. All four tested *Luffa* species showed maximum pollen viability percentage (>95%) using both staining methods, with no significant (*p* ≤ 0.05) difference among the species (**Figures 4**, **5**). An overestimation of staining tests (KI and AC) in pollen viability assessment over *in vitro* germination was observed in all four species tested.

### Pollen moisture content and desiccation period

**Figure 6:**
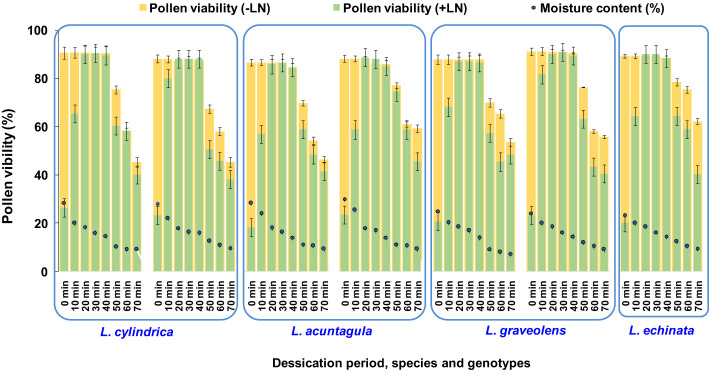
Determination of optimum pollen moisture content (MC) and desiccation period for cryopreservation of *Luffa* species pollen. Data presented in vertical bars represent mean for pollen viability (-LN and +LN). Circular dots represent mean for pollen moisture content.

The fresh pollen moisture content ranged from 23.09% to 28.98% among the seven genotypes of the four species studied (**Figure 6**). Among the different desiccation periods (ranging from 10 to 60 min) tested, regardless of genotype, the highest pollen viability after cryopreservation was reported after 20, 30, and 40 min, resulting in an increase (0.17% to 0.23%) and a decrease (0% to 2.67%) compared to the respective fresh pollen viability. A significant increase in pollen viability after cryopreservation was observed as the desiccation period increased from 0 to 40 min. However, a further increase in the desiccation period to 50 and 60 min resulted in a decrease in pollen viability. Thus, desiccation for 20 to 40 min, attaining a moisture content of 14.04% to 18.55%, was found to be most effective and was applied in subsequent experiments.

### Thawing temperature

**Figure 7:**
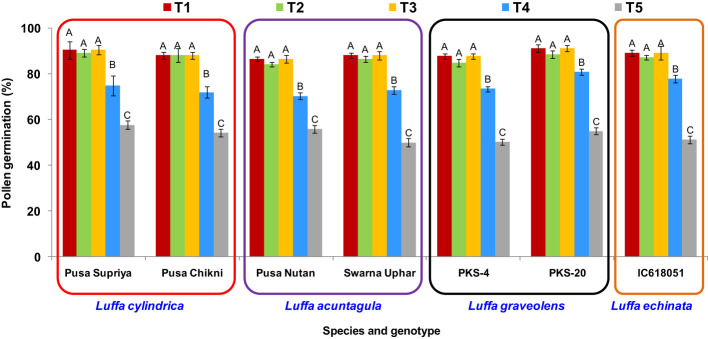
Effect of thawing methods on post-thaw germination of cryopreserved pollen in *Luffa* species. (T1) thawing at room temperature of 25 °C (±2°C) for 20 min., (T2) thawing under running water at 20 °C (±5°C) for 10 min., (T3) thawing in water held at 35 °C for 5 min. with constant slow stirring, (T4) thawing under 10 °C for 5 min., and (T5) thawing under 10 °C for 2 min. followed by thawing at 35 °C for 3 min. Data presented in vertical bars represent mean ± SE. Significant differences (P ≤ 0.05) in pollen viability are presented by different alphabets analyzed by Duncan’s Multiple Range Test.

Irrespective of the genotype tested, the highest post-thaw pollen viability was observed when using three different methods: thawing at 20°C (± 5°C), 25°C ( ± 2°C), and 35°C. Significant declines in post-thaw pollen viability were observed with the other two thawing methods (thawing under 10°C for 5 min, and 10°C for 2 min followed by thawing at 35°C for 3 min) (**Figure 7**). Therefore, for ease of operation, thawing at room temperature of 25°C ( ± 2°C) for 20 min was used in the subsequent experiments.

### Pollen storage

**Figure 8:**
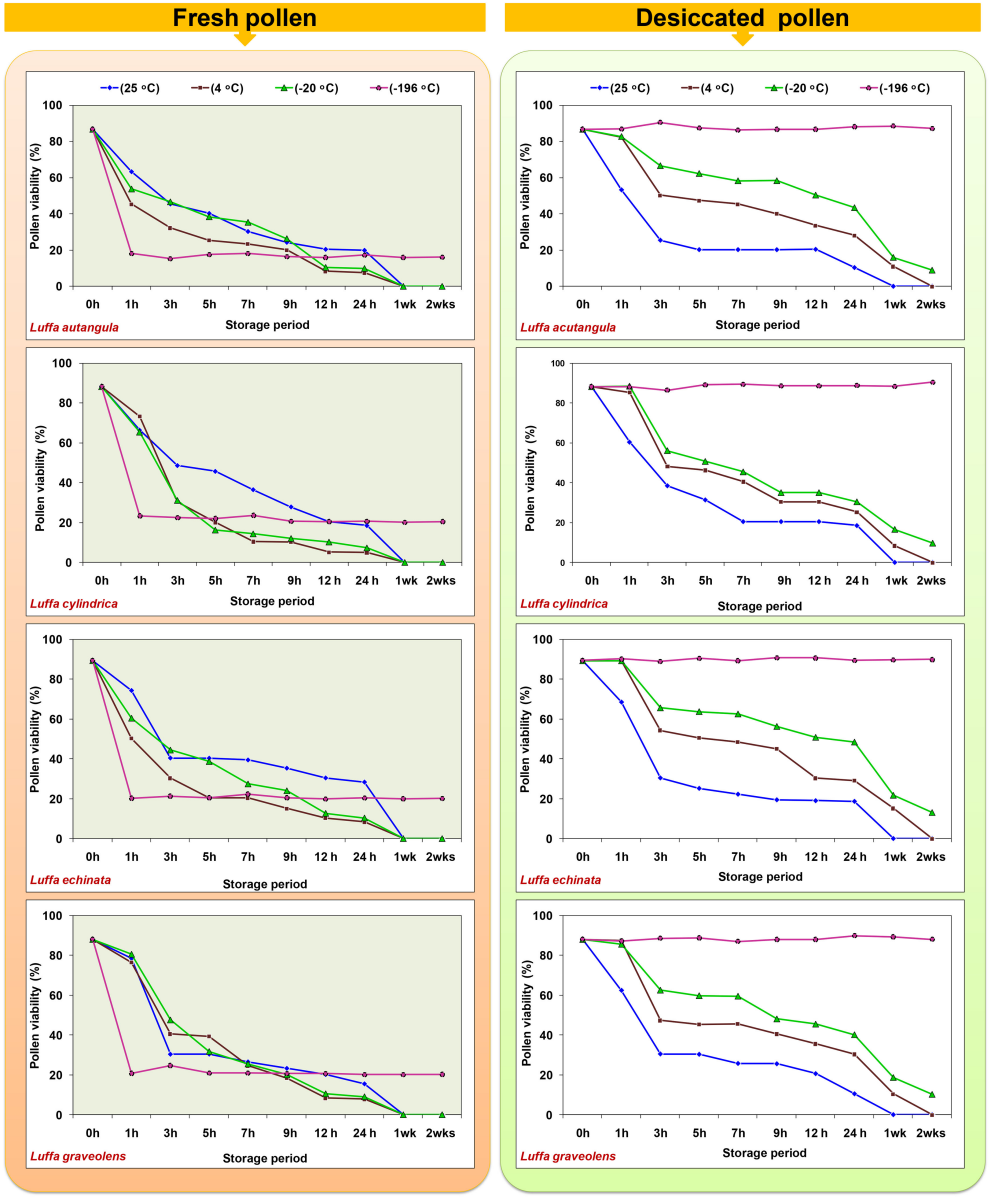
Effect of storage temperature and duration on pollen viability of fresh and desiccated pollen in *Luffa* species.

**Figure 9:**
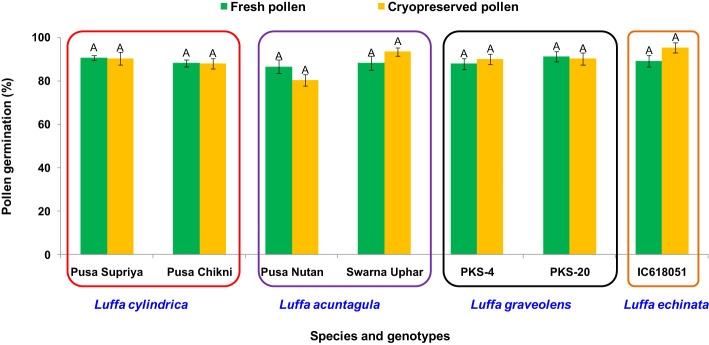
Post-thaw viability of cryopreserved pollen stored for 2 months in *Luffa* species. Data presented in vertical bars represent mean ± SE. Significant differences (*P* ≤ 0.05) in pollen viability are presented by different alphabets analyzed by Duncan’s Multiple Range Test.

In the present study, both fresh and desiccated pollen of four *Luffa* species were stored at various temperatures (25, 4, −20, and −196°C) for a period of 2 weeks (**Figure 8**). In all tested *Luffa* species, significant variation in pollen viability was observed between fresh and desiccated pollen at room temperature across all the storage periods. Fresh pollen exhibited greater pollen viability over time compared to desiccated pollen. Both fresh and desiccated pollen began to lose viability gradually after 1 h of storage and completely lost viability after 24 h of storage (**Figure 8**). In contrast to room temperature storage, desiccated pollen showed higher viability than fresh pollen when stored at 4°C and −20°C. After 1 week of storage, desiccated pollen showed approximately 10% pollen viability, while fresh pollen completely lost its pollen viability after 1 week at 4°C. A similar trend of fresh pollen viability at different storage periods and temperatures was also observed at −20°C, where pollen completely lost its viability after 1 week. Meanwhile, desiccated pollen remained viable for up to 2 weeks with approximately 9% to 13% pollen viability. Similar to storage at room temperature, 4°C, and −20°C, fresh pollen (not desiccated) stored at −196°C resulted in a >70% decline in pollen viability compared to fresh pollen viability. In contrast, desiccated pollen retained viable for 2 months, with no significant variation in pollen viability between fresh and desiccated cryopreserved pollen (**Figure 9**).

### Fertilizing ability

**Figure 10:**
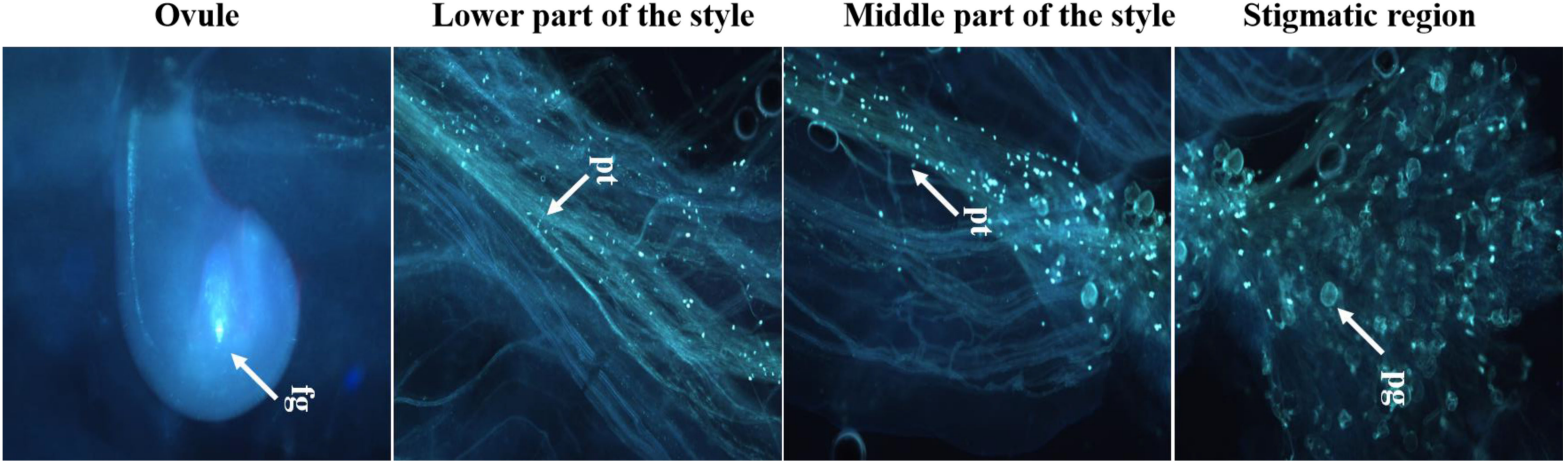
Fertilizing ability of cryopreserved pollen on receptive stigma using fluorescence microscopy; pg, pollen germination; pt, pollen tube growth; fg, fertilized ovule; Scale bar = 200 µm.

**Figure 11:**
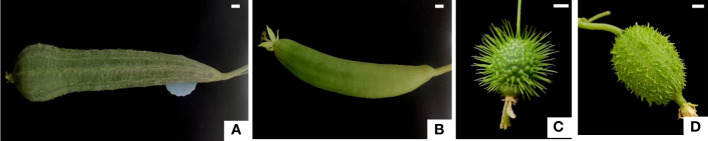
Fertilizing ability of cryopreserved pollen using field pollinations. **(A–D)** Fruit set using cryopreserved pollen, **(A)** *L. cylindrica*, **(B)** *L. acutangala*, **(C)** *L. echinata*, and **(D)** *L. graveolens*. Scale bar = 1 cm.

Both fresh (control) and cryopreserved pollen exhibited similar patterns of pollen germination and pollen tube development (**Figure 10**). Pollen germinated on the receptive stigmatic surface at 2–4 HAP, entered the pollen tube and embryo sac through the micropyle, and fertilized the ovule within 12 HAP. Pollen–pistil behavior showed approximately 92.24% pollen germination in both fresh and cryopreserved pollen, with no significant variation noticed at 12, 24, and 48 HAP. Also, no significant variation in fruit set (%), fruit length (cm), fruit width (cm), average fruit weight (g), number of seeds per fruit, seed length (mm), and seed width (mm) (**Table 1**) (**Figures 11A–D**) was observed between the flowers pollinated with cryopreserved pollen and fresh pollen, irrespective of the species.

**Table 1:** Fruit and seed characters in crosses using fresh and cryopreserved pollen in *Luffa* species.

Parameters	Fruit set (%)		Fruit length (cm)		Fruit width (cm)		Average fruit weight (g)		No. of seeds per fruit		Seed length (mm)		Seed width (mm)	
	(−LN)	(+LN)	(−LN)	(+LN)	(−LN)	(+LN)	(−LN)	(+LN)	(−LN)	(+LN)	(−LN)	(+LN)	(−LN)	(+LN)
Pusa Supriya	94.7 ± 1.4^a^	94.4 ± 1.3^a^	21.9 ± 0.79^a^	20.9 ± 1.3^a^	3.0 ± 0.1^a^	3.1 ± 0.1^a^	151.7 ± 3.0^a^	148.5 ± 3.6^a^	93.2 ± 1.5^a^	90.4 ± 2.9^a^	13.1 ± 0.1^a^	13.4 ± 0.1^a^	7.9 ± 0.2^a^	7.9 ± 0.2^a^
Pusa Chikni	93.2 ± 1.8^a^	95.7 ± 0.5^a^	22.4 ± 1.2^a^	22.8 ± 1.4^a^	3.2 ± 0.1^a^	2.9 ± 0.8^a^	149.03 ± 2.0^a^	152.4 ± 2.9^a^	93.7 ± 1.5^a^	90.2 ± 2.4^a^	12.1 ± 0.2^a^	12.1 ± 0.4^a^	7.1 ± 0.1^a^	7.0 ± 0. ^a^
Pusa Nutan	90.4 ± 1.5^a^	88.5 ± 1.2^a^	26.8 ± 1.7^a^	26.5 ± 1.1^a^	3.4 ± 0.1^a^	3.3 ± 0.4^a^	130.4 ± 2.8^a^	135.4 ± 2.5^a^	84.4 ± 0.5^a^	83.8 ± 0.9^a^	12.3 ± 0.2^a^	11.9 ± 0.8^a^	6.4 ± 0.3^a^	6.3 ± 0.5^a^
Swarna Uphar	92.6 ± 1.4^a^	90.4 ± 1.9^a^	21 ± 1.7^a^	22.1 ± 1.8^a^	3.8 ± 0.1^a^	3.4 ± 0.6^a^	119.8 ± 3.0^a^	118.4 ± 2.4^a^	83.4 ± 0.7^a^	80.5 ± 0.9^a^	12.0 ± 0.2^a^	12.2 ± 0.6^a^	7.0 ± 0.1^a^	6.9 ± 0.9^a^
PKS-4	94.5 ± 1.3^a^	95.6 ± 1.4^a^	5.1 ± 0.1^a^	4.9 ± 0.4^a^	2.7 ± 0.1^a^	2.8 ± 0.1^a^	7.6 ± 1.3^a^	7.4 ± 1.4^a^	31.1 ± 0.6^a^	32.3 ± 0.1^a^	9.7 ± 0.5^a^	9.3 ± 0.5^a^	5.6 ± 0.3^a^	5.7 ± 0.7^a^
PKS-20	95.2 ± 1.8^a^	93.7 ± 2.0^a^	4.8 ± 0.2^a^	5.2 ± 0.5^a^	2.4 ± 0.1 ^a^	2.3 ± 0.2^a^	7.8 ± 0.2^a^	7.7 ± 1.0^a^	37.3 ± 2.6^a^	36.4 ± 0.9^a^	10.6 ± 0.3^a^	10.5 ± 0.3^a^	5.9 ± 0.1^a^	5.9 ± 0.2^a^
IC618051	95.7 ± 1.6^a^	91.3 ± 2.4^a^	3.7 ± 0.3^a^	3.84 ± 0.6^a^	2.0 ± 0.1^a^	2.1 ± 0.1^a^	3.8 ± 0.3^a^	3.5 ± 0.8^a^	20.4 ± 1.7^a^	20.2 ± 0.2^a^	6.7 ± 0.4^a^	6.5 ± 0.6^a^	4.1 ± 0.1^a^	4.0 ± 0.3^a^

Data presented in table represent mean ± SE. Significant differences (p ≤ 0.05) in each trait among the genotypes are presented by different letters analyzed by Duncan’s Multiple Range Test. (−LN) represents fresh pollen and (+LN) represents cryopreserved pollen.

## Discussion

Effective and successful pollination is a pre-requisite for seed and fruit production in most plants, and knowledge on anthesis and pollen viability is essential for better planning of hybridization programs to increase the success of crop improvement. The optimum time for pollen collection is 5:00 and 6:00 PM in *L. acutangula* and 8:00 to 9:00 AM in *L. cylindrica*, *L. echinata*, and *L. graveolens*, when flowers open freshly and anther dehiscence occurs. Pollen viability is a key indicator of pollen quality and can be assessed by *in vivo* and *in vitro* techniques, including (a) seed set percentage in field, (b) staining techniques (KI, TTC, acetocarmine, and fluorescein diacetate), (c) pollen–pistil studies in a fluorescence microscope, and (d) *in vitro* germination (Dafni and Firmage, 2000). Since it mimics *in vivo* settings, *in vitro* pollen germination is one of the most efficient and reliable ways to assess the viability of the pollen (Sakhanokho and Rajasekaran, 2010; Pereira et al., 2018; Aldahadha et al., 2019; Luo et al., 2020). BK is the most commonly used medium for pollen germination in several species, including *Momordica* species (Rathod et al., 2018), *Abelmoschus* species (Gowthami et al., 2021; Gowthami et al, 2023), *Annona squamosa* L. (Chander et al., 2019), *Psidium* species (Vishwakarma et al., 2021), *Ocimum* species (Kumari et al., 2022), and *Lathyrus sativus* (Shankar et al., 2023).

Sucrose plays a major role in quick germination, percent pollen germination, and pollen tube development, providing energy and maintaining osmotic balance between pollen grains and the culture medium (Lin et al., 2017; Fragallah et al., 2019; Gowthami et al., 2021). BK medium with 10% sucrose reported significantly (*p* ≤ 0.05) higher fresh pollen germination compared to the rest of the sucrose concentrations in *L. cylindrica* (88.15% to 90.61%), *L. acutangula* (86.66% to 88.17%), and *L. echinata* (89.29%), while BK medium with 3% sucrose was found to be optimum for *L. graveolens* (87.84 to 91.19%) (**Figure 2**). This justifies the species-specific requirement for sucrose concentration. Similar species-dependent sucrose concentration trends were also reported by Soares et al, 2013; Soares et al, 2015, dos Santos Ferreira et al. (2021), and Anushma and Rajasekharan (2023). A positive relationship between sucrose concentrations and pollen germination was observed in *L. acutangula*, *L. cylindrica*, and *L. echinata*, with gradual increase in pollen germination as sucrose concentration increased from 0% to 10%. Further increases in sucrose concentration to 15%, 20%, and 30% had a negative effect and led to the decreased pollen germination, along with reduced germination, bursting of pollen grains, and malformed pollen tubes. These differences in pollen germination percentage and media composition among the different species are likely related to the variable energy demands of the species, requiring careful tailoring of sucrose concentration in the culture medium for each species. Incubation temperature is a factor that influences *in vitro* pollen germination (Boavida and McCormick, 2007; Lin et al., 2017). The optimum incubation temperature for pollen germination in all the species was 25°C (**Figure 3**). Similar results were also reported by Sukhvibul et al. (2000), where 25°C was found to be optimum for mango pollen germination.

Staining tests are widely used for determining pollen viability because they are quick and easy (Huang et al., 2004; Li et al., 2023). The assessment of pollen viability by staining tests (KI and AC) in the present study revealed that >95% pollen viability was observed in all species, with an overestimation of 6%–10% in AC staining and 7%–10% in KI staining in comparison to *in vitro* pollen germination (**Figure 4**). Although staining tests are widely used, overestimation of pollen viability in comparison to *in vitro* pollen germination was reported by several authors, including Gowthami et al. (2021) in *A. moschatus* and Shankar et al. (2023) in *L. sativus*.

The moisture content of pollen is one of the critical factors in determining the post-storage viability during low and ultra-low temperatures, which varies with the developmental stage and mature stage of pollen during dispersal, including bicellular and tricellular pollen (Bajaj, 1987). Bicellular pollen (desiccation-resistant, orthodox pollen) can typically withstand desiccation up to 11.1% moisture content (Towill, 1985), and pollen viability is decreased by water loss below 10% moisture content. Pollen with high initial moisture content (35%–40%) is sensitive to low temperatures (Dafni and Firmage, 2000; Rajasekharan and Ravi, 2023), causing freezing of water inside the cells and leading to the formation of ice crystals. These ice crystals damage pollen membranes and cause alterations in the osmotic, structural, and colligative integrity of cells, resulting in mechanical and physical injury that ultimately leads to viability loss (Towill, 1981; Xu et al., 2014; de Souza et al., 2015; Liu et al., 2015). Pre-drying pollen to attain optimum moisture content before ultra-low temperature storage is essential to maintaining the viability of pollen (Nepi et al., 2001; Pacini and Hesse, 2004; Dinato et al., 2020), as it results in the formation of a glassy state quickly and avoids ice crystal formation. Thus, desiccation for 20 to 40 min, attaining a moisture content of 14.04% to 18.55%, was found to be most effective in the present study.

Thawing after cryostorage also significantly influences pollen viability (Sauve et al., 2000; He et al., 2017; Jia et al., 2022). Thawing can be achieved through either rapid or slow thawing processes, and the choice of thawing method greatly affects the cell membrane system, water absorption, subsequent freezing, and osmotic shock of water (Zhang et al., 2006). Among the different thawing temperatures tested for cryopreserved pollens, the highest post-thaw pollen viability was consistently observed using three different methods: thawing at 20°C ( ± 5°C), 25°C ( ± 2°C), and 35°C (**Figure 7**).

Temperature and humidity play pivotal roles in determining pollen viability during storage (Ren et al., 2019). At room temperature (25°C), pollen stored for 1 week did not exhibit any viability, whether fresh or desiccated. This lack of viability may be attributed to the combination of high temperature, humidity, heightened respiration and metabolism, severe water loss, and rapid decline in vitality all contributing to poor storage characteristics (Akond et al., 2012; Du et al., 2019). In contrast, storage of desiccated pollen grains at low temperatures, specifically 4°C and −20°C, extended pollen longevity 1 week (10% viability) and up to 2 weeks (9%–13% viability), respectively (**Figure 8**). The storage at 4°C likely inhibits pollen metabolic activity, resulting in a gradual reduction and eventual total loss of viability. On the other hand, storage at −20°C further slows down physical and chemical processes within the pollen, leading to a loss of pollen viability or death (Akond et al., 2012). For long-term preservation, cryopreservation at −196°C is recommended, ensuring high pollen viability for up to 2 months (**Figures 8**, **9**). Importantly, cryopreserved pollen maintains its original viability with only slight fluctuations, which are statistically non-significant, when compared to fresh pollen (**Figure 8**, **9**). Pollen cryopreservation serves as an essential tool in managing plant genetic resources for the long-term conservation of haploid genetic resources to support plant breeding efforts (Dinato et al., 2020).

*In vivo* pollen germination and pollen tube growth of cryopreserved pollen on receptive stigmas, studied in *L. acutangula* using fluorescence microscopy, revealed that both fresh (control) and cryopreserved pollen exhibited similar pattern of pollen germination and pollen tube development. Similar observations were also reported in previous studies, such as those on *A. moschatus* (Gowthami et al., 2021) and *L. sativus* (Shankar et al., 2023). Furthermore, for a more reliable assessment, field pollinations were conducted, and no negative effect of cryopreservation on ovule fertilization, and fruit and seed set was observed after pollination with cryopreserved pollen. These observations align with earlier reports on various crops, including *Colocasia esculenta* (Mukherjee et al., 2016), *Manihot esculenta* (Hegde et al., 2019), mango, and litchi (Chaudhury et al., 2010), confirming the effectiveness and reliability of pollen cryopreservation techniques for maintaining plant genetic resources and supporting breeding programs.

## Conclusion

This study optimized pollen germination mediums and conditions for *Luffa* species, identifying BK medium with 10% sucrose for *L. acutangula*, *L. cylindrica*, and *L. echinata*, and BK medium with 3% sucrose for *L. graveolens*, all incubated at room temperature. Fresh pollen storage was limited to 24 h at room temperature, with viability ranging from 10% to 18%. In contrast, desiccated pollen showed improved storage, maintaining the viability for 1 week at 4°C (10% viability) and for 2 weeks at −20°C (9%–13% viability). For long-term preservation, cryopreservation at −196°C retained high viability for up to 2 months. Desiccation periods of 20, 30, and 40 min achieved optimum moisture content (14.04% to 18.55%) for successful cryopreservation. Both *in vitro* and *in vivo* pollen assessments of cryopreserved pollen demonstrated similar results to fresh pollen, confirming the protocol’s reliability. This method holds promise for facilitating hybridization and gene transfer in *Luffa* species, enhancing resilience to various stresses, and managing plant genetic resources effectively.

## Data availability statement

The original contributions presented in the study are included in the article. Further inquiries can be directed to the corresponding authors.

## Author contributions

AN: Conceptualization, Investigation, Writing – original draft. RG: Conceptualization, Resources, Supervision, Visualization, Writing – original draft, Writing – review & editing. ASu: Writing – original draft, Writing – review & editing, Resources, Supervision, Visualization. AM: Resources, Supervision, Visualization, Writing – review & editing. MV: Supervision, Visualization, Writing – original draft, Writing – review & editing. ASi: Writing – review & editing, Data curation, Supervision. NM: Writing – review & editing, Data curation, Resources, Supervision. JS: Writing – review & editing, Data curation. SC: Writing – original draft. MS: Writing – review & editing, Investigation. PP: Writing – review & editing, Investigation, Supervision. SR: Writing – review & editing, Data curation, Supervision.
